# Co-utilization of L-arabinose and D-xylose by laboratory and industrial *Saccharomyces cerevisiae *strains

**DOI:** 10.1186/1475-2859-5-18

**Published:** 2006-04-10

**Authors:** Kaisa Karhumaa, Beate Wiedemann, Bärbel Hahn-Hägerdal, Eckhard Boles, Marie-F Gorwa-Grauslund

**Affiliations:** 1 Department of Applied Microbiology, Lund University, P.O. Box 124, SE-22100 Lund, Sweden; 2 Institute of Molecular Biosciences Goethe-University Frankfurt am Main, Marie-Curie-Str. 9, D-60439 Frankfurt am Main, Germany

## Abstract

**Background:**

Fermentation of lignocellulosic biomass is an attractive alternative for the production of bioethanol. Traditionally, the yeast *Saccharomyces cerevisiae *is used in industrial ethanol fermentations. However, *S. cerevisiae *is naturally not able to ferment the pentose sugars D-xylose and L-arabinose, which are present in high amounts in lignocellulosic raw materials.

**Results:**

We describe the engineering of laboratory and industrial *S. cerevisiae *strains to co-ferment the pentose sugars D-xylose and L-arabinose. Introduction of a fungal xylose and a bacterial arabinose pathway resulted in strains able to grow on both pentose sugars. Introduction of a xylose pathway into an arabinose-fermenting laboratory strain resulted in nearly complete conversion of arabinose into arabitol due to the L-arabinose reductase activity of the xylose reductase. The industrial strain displayed lower arabitol yield and increased ethanol yield from xylose and arabinose.

**Conclusion:**

Our work demonstrates simultaneous co-utilization of xylose and arabinose in recombinant strains of *S. cerevisiae*. In addition, the co-utilization of arabinose together with xylose significantly reduced formation of the by-product xylitol, which contributed to improved ethanol production.

## Introduction

Large interest in biofuel ethanol production from renewable sources has led in numerous attempts to construct microbial strains that efficiently ferment lignocellulose hydrolysate to ethanol [1,2]. The optimal micro-organism must be robust, ethanol and inhibitor tolerant, viable at low pH, and produce high ethanol yields from all sugars present in lignocellulose [3]. The baker's yeast *Saccharomyces cerevisiae *is very suitable for industrial ethanol production, however, it cannot utilize pentoses that represent a significant fraction of the sugars present in lignocellulosic raw materials. As examples, 16% xylan and 5% arabinan are found in grass, 19% xylan and 3% arabinan are found in corn stover, and as high as 15% arabinan and 19% xylan are found in wheat bran [4]. Utilization of all available substrate is a prerequisite for an economically feasible process, and even small increases in substrate utilization may significantly improve the overall process [5].

Several genetic engineering strategies have been used in attempt to enable xylose or arabinose utilization by *S. cerevisiae*. Xylose fermentation has been achieved by expressing *Pichia stipitis *xylose reductase (XR) and xylitol dehydrogenase (XDH) together with overexpression of the endogenous xylulokinase (XK) [6-8]. Xylose growth and fermentation have also been achieved by expressing xylose isomerase from bacterial [9] or fungal [10] sources. Arabinose utilization in *S. cerevisiae *has been enabled by expressing bacterial [11] or fungal [12] arabinose pathways. Efficient growth and fermentation of arabinose was achieved with high-level expression of the bacterial arabinose pathway consisting of *Bacillus subtilis *L-arabinose isomerase (*AraA*), *Escherichia coli *L-ribulokinase (*AraB*) and *E. coli *L-ribulose-5-P 4-epimerase (*AraD*), together with the endogenous pentose-transporting permease gene *GAL2*, followed by selection for improved arabinose growth by sequential transfer in arabinose medium [11].

To the best of our knowledge, no *S. cerevisiae *strain with demonstrated co-utilization of xylose and arabinose has previously been developed. *E. coli *is naturally able to utilize both xylose and arabinose, and it has been engineered to redirect the product formation towards ethanol [13]. Inversely, co-utilization of xylose and arabinose was achieved in the ethanol-producing bacterium *Zymomonas mobilis *by introducing the bacterial arabinose and xylose utilization pathways [14]. However, the industrial use of bacteria for the fermentation of lignocellulose hydrolysates remains problematic due to their low tolerance towards inhibitors [15].

In this work, we developed and characterized yeast strains co-consuming and co-fermenting both pentose sugars. The XR, XDH and XK activities together with the bacterial arabinose pathway were first introduced in a *S. cerevisiae *laboratory CEN.PK strain [16] to demonstrate the functional co-utilization of the two pentoses. A method for multiple integration of genes in the ribosomal DNA (rDNA) [17] was applied to construct stable high-level expression of the arabinose genes in industrial yeast strains. Using this tool, an arabinose and xylose utilizing industrial strain based on the xylose-fermenting strain TMB 3400 [18] was developed.

## Results

### Development of a *S. cerevisiae *laboratory strain co-consuming D-xylose and L-arabinose

To demonstrate functional co-utilization of xylose and arabinose by recombinant yeast, the metabolic pathways for both xylose and arabinose utilization were expressed in a *S. cerevisiae *laboratory strain. The construct was based on the arabinose-fermenting strain JBY25-4M [11] (Table 1), which contains the bacterial arabinose pathway consisting of the genes for L-arabinose isomerase (*AraA*), L-ribulokinase (*AraB*) and L-ribulose-5-P 4-epimerase (*AraD*) as well as the overexpressed endogenous galactose transporter gene (*GAL2*), each on a separate multicopy plasmid, and which also has beneficial mutations resulting from prolonged culturing in arabinose medium [11]. One mutation resulted in the amplification of the *TAL1 *gene [11], but other unknown mutations are possibly also present. Because the strain JBY25-4M can retain the four plasmids only in the presence of a selection pressure for arabinose utilization, the plasmids of JBY25-4M were removed by cultivation in rich medium before further genetic modification. In the resulting strain, the xylose pathway genes were introduced using the integrative vector YIpXR/XDH/XK [7] (Table 1) which contains the *P. stipitis *xylose pathway genes coding for XR (*XYL1*), XDH (*XYL2*) and the endogenous XK (*XKS1*), selecting for histidine prototrophy. The resulting strain was named BWY02.X. Presence of the functional xylose pathway was verified by xylose growth. A prototrophic control strain without the arabinose pathway was constructed by introducing three empty plasmids, p424H7, p425H7, and p426H7 [11] (Table 1) without the arabinose genes into BWY02.X, resulting in strain BWY02.Xp. To generate a xylose- and arabinose-consuming strain, three multicopy plasmids carrying the three bacterial arabinose pathway genes were then introduced in BWY02.X. *B. subtilis AraA *gene, mutated *E. coli AraB *gene and *E. coli AraD *gene were introduced in BWY02.X. using plasmids YEpURA*araA *(Table 1), YEp*araB *[11] and YEp*araD *[11], respectively, selecting for prototrophy and growth on arabinose. The resulting strain was named BWY02.XA.

**Table 1:** *S. cerevisiae *strains and plasmids used in this study.

*S. cerevisiae *strains	Relevant genotype/phenotype	Reference
JBY25-4M	*MATa leu2-3,112 ura3-52 trp1-289 his3-Δ1 MAL2-8*^*c *^*SUC2, unknown beneficial mutations for arabinose utilization*, with plasmids YEp*araA*, YEp*araB*^*G361A*^, YEp*araD*, YEp*GAL2*	[11]
BWY02.X	*MATa leu2-3,112 ura3-52 trp1-289 his3-Δ1 MAL2-8*^*c *^*SUC2*, *unknown beneficial mutations for arabinose utilization*, *HIS3*::YIpXR/XDH/XK	This work
BWY02.Xp	BWY02.X with plasmids p424H7, p425H7, p426H7	This work
BWY02.XA	BWY02.X with plasmids YEpURAaraA, YEp*araB*^*G361A*^, YEp*araD*	This work
TMB 3400	*HIS3*::pPGK-*Xyl1*-tPGK, pADH-*Xyl2*-tADH, pPGK-*XKS1*-tPGK	[18]
TMB 3060	TMB 3400, *TRP1*::YIpAraB KanMX	This work
TMB 3061	TMB 3060, *NTS2::pHXT7-AraA-tCYC1*and *NTS2::pHXT7-AraD-tCYC1*, KanMX	This work
TMB 3063	TMB 3061 retransformed with *NTS2::pHXT7-AraA-tCYC1*	This work
CEN.PK 113-7D	*MATa *(prototrophic)	[16]
*Plasmids*		
YIpXR/XDH/XK	*pPGK1-XYL1-tPGK1, pADH1-XYL2-tADH1, pPGK1-XKS1-tPGK1, HIS3*	[7]
YEp*araA*	*pHXT7-AraA-tCYC1, HIS3*	[11]
YEpURAaraA	*pHXT7-AraA-tCYC, URA3*	This work
YEp*araB*	*pHXT7-AraB-tCYC1*, *TRP1*	[11]
YEp*araD*	*pHXT7-AraD-tCYC1, LEU2*	[11]
p423H7	*pHXT7-MCS-tCYC1, HIS3*	[11]
p424H7	*pHXT7-MCS-tCYC1, TRP1*	[11]
p425H7	*pHXT7-MCS-tCYC1,, LEU2*	[11]
p426H7	*pHXT7-MCS-tCYC1, URA3*	[11]
pBluescript II SK	Cloning vector	(Stratagene, La Jolla, CA, USA)
YIpAraB	*KanMX, pHXT7-AraB-tCYC1 TRP1*	This work
prDNA	pBluescript, *NTS2*	This work
prDNAAraA	prDNA, *NTS2::pHXT7-AraA-tCYC1*	This work
prDNAAraD	prDNA, *NTS2::pHXT7-AraD-tCYC1*	This work

**Figure 1:**
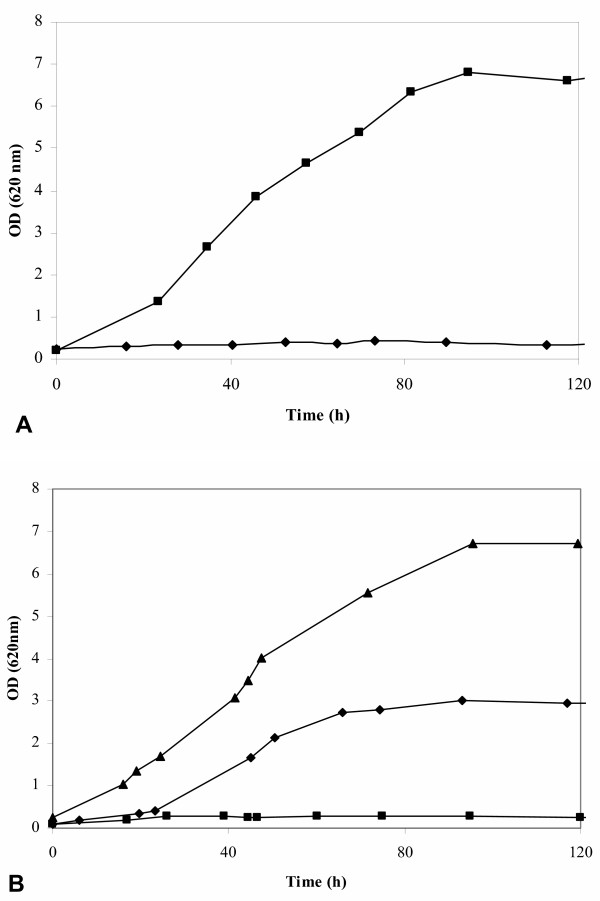
Aerobic growth of *S. cerevisiae *strains in YNB medium with 50 g/l arabinose as the sole carbon source. **A**. Laboratory strains BWY02.Xp (◆) (xylose pathway and empty multicopy plasmids) and BWY02.XA (■) (xylose and arabinose pathways). **B**. Industrial strains TMB 3400 (■) (xylose pathway), TMB 3061 (◆)(xylose and arabinose pathways), and TMB 3063 (▲) (xylose and arabinose pathways).

The functional xylose pathway in BWY02.XA was verified by xylose growth. BWY02.XA grew aerobically on 50 g/l xylose with a growth rate of 0.013 h^-1 ^(data not shown). Similar *in vitro *activities of XR and XDH were obtained for BWY02.XA and the xylose-growing control strain TMB3001 [7]. For BWY02.XA, the XR activity was 0.08 ± 0.01 U mg protein^-1 ^and the XDH activity was 1.36 ± 0.07 U mg protein^-1^, whereas the activities for TMB 3001 were 0.10 ± 0.01 U mg protein^-1 ^and 1.01 ± 0.08 U mg protein^-1^, respectively. Very low activities of 0.007 ± 0.002 U mg protein^-1 ^(XR) and 0.004 ± 0.001 U mg protein^-1 ^(XDH) were measured in the negative control strain CEN.PK113-7D. The presence of an active arabinose pathway in BWY02.XA was demonstrated by aerobic growth on arabinose. *In vitro *activities for the arabinose pathway enzymes could not be measured due to the reagents required not being commercially available. BWY02.XA grew in aerobic shake-flask cultures with 50 g/l arabinose at a growth rate of 0.05 h^-1 ^until an OD_620 nm _of about 6, whereas the control strain BWY02.Xp did not grow on arabinose (Figure 1A). BWY02.XA utilized arabinose and xylose simultaneously in aerobic shake-flask cultures containing a mixture of 50 g/l xylose and 50 g/l arabinose. Arabinose was the preferred substrate, with 35% of the arabinose and 15% of the xylose being consumed within 95 hours (data not shown).

### Genetic tools for stable genomic integration of the arabinose pathway genes in industrial *S. cerevisiae *strains

**Figure 2:**
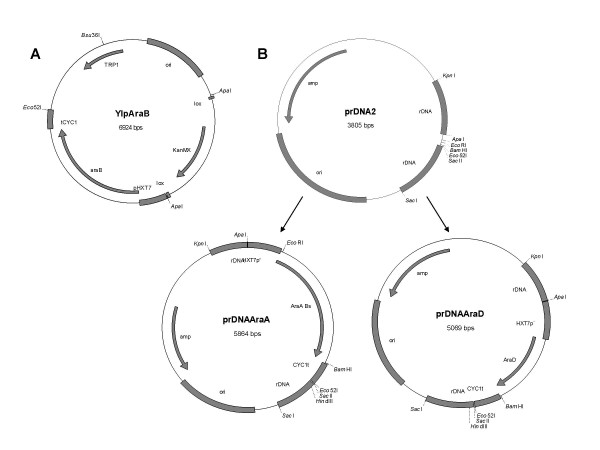
Plasmids used in construction of the industrial strains TMB 3061 and TMB 3063. **A**. Integrative vector YIpAraB. **B**. Multicopy plasmid prDNA2 and the derivative plasmids prDNAAraA and prDNAAraD that are used as templates for PCR-amplification.

Previously, the best arabinose utilization by a laboratory strain has been obtained by expressing the *AraA *and *AraD *genes from multi-copy plasmids and the L-ribulokinase gene *AraB *either in a mutated form with reduced activity or from a single copy centromeric vector [11]. In order to reproduce this pattern in industrial yeast strains, genetic tools aiming at multiple chromosomal integration of the *AraA *and *AraD *genes were constructed. The rDNA region of the *S. cerevisiae *genome was chosen as target for multiple integration because the rDNA region is present at about 140 copies in the yeast genome [19] and the strategy has previously been successfully applied [20-22]. Plasmid prDNA2 (Figure 2B) containing two adjacent 500 bp long sequences from the *S. cerevisiae *rDNA was first constructed (see Materials and Methods). The *AraA *and *AraD *genes were separately cloned in prDNA2, resulting in two plasmids prDNA-AraA and prDNA-AraD (Figure 2B), respectively. The plasmids were then used as a template for creating PCR-fragments to be used for transformation, consisting of either of the genes flanked by the rDNA-sequences. Instead of the more commonly used linearized plasmids, PCR-fragments were preferred for yeast transformation to avoid introducing unnecessary antibiotic markers or other vector sequences, as well as to keep the introduced sequence as short as possible.

### Construction of a stable industrial *S. cerevisiae *strain co-consuming xylose and arabinose

The chromosomal integration of the arabinose pathway genes was applied on the xylose-fermenting industrial strain TMB 3400 [18]. First, single integration of the mutated *E. coli *L-ribulokinase gene *AraB *[11] was performed on TMB 3400 using the vector YIpAraB (Figure 2A) linearized by *Eco81I*, resulting in integration in the *TRP1*-locus. Transformants were selected on geneticin, and the presence of the *AraB *gene was confirmed by PCR. The resulting yeast strain was named TMB 3060. For multiple integration, the *AraA *and *AraD *genes flanked by the rDNA target sequences were PCR-amplified from plasmids prDNA-AraA or prDNA-AraD (Figure 2B) and both PCR fragments were transformed simultaneously into TMB 3060. Since introduction of these two genes completed the arabinose utilization pathway, the transformants were selected for growth on arabinose. One transformant was purified and the resulting strain was named TMB 3061.

**Figure 3:**
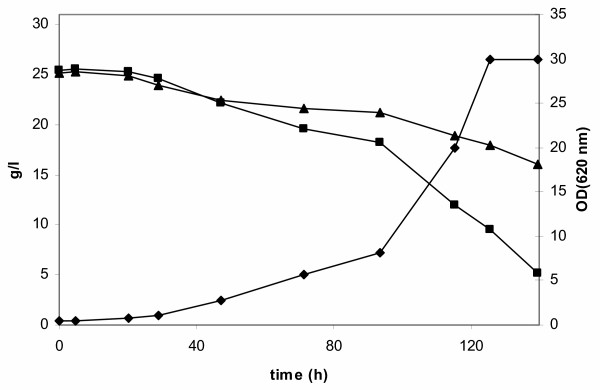
Growth of *S. cerevisiae *TMB 3061 (xylose and arabinose-growing industrial strain) in mineral medium containing a mixture of 25 g/l arabinose and 25 g/l xylose in aerobic batch culture and co-consumption of arabinose and xylose. Left axis: arabinose (▲), xylose (■) (g/l), right axis: OD_620 _(◆).

To verify functional arabinose utilization in TMB 3061, the aerobic arabinose growth rates of TMB 3400 and TMB 3061 were compared. TMB 3061 grew with a maximum specific growth rate of 0.06 h^-1 ^in YNB medium with 50 g/l arabinose, whereas the parental strain TMB 3400 did not grow on arabinose (Figure 1B). Growth of TMB 3061 stopped at an OD_620 nm _of about 3, indicating presence of a limiting step in the existing metabolic pathway. In aerobic shake-flask cultures using YNB medium with a mixture of 25 g/l xylose and 25 g/l arabinose as carbon sources, TMB 3061 consumed arabinose and xylose simultaneously, xylose being the preferred sugar (Figure 3). No arabinose consumption, but nearly complete xylose consumption was observed for TMB 3400 (data not shown).

In order to further improve arabinose utilization, TMB 3061 was re-transformed with the *AraA *gene encoding L-arabinose isomerase, since previous work on JBY25-4M showed that the number of the plasmid carrying the *AraA *gene exceeded the numbers of the other plasmids, indicating that L-arabinose isomerase activity may be limiting for growth on arabinose [11]. Transformants with improved arabinose growth were selected in liquid YNB medium containing 20 g/l arabinose. After three rounds of sequential transfer to new liquid cultures, aliquots were plated on arabinose plates and single colonies were purified and analyzed for arabinose growth. The best clone was named TMB 3063. Strain TMB 3063 grew on 50 g/l arabinose with a slightly reduced growth rate of 0.04 h^-1^, but reached a significantly increased final OD_620 nm _of 6 (Figure 1B), which is the same as for the laboratory strain BWY02.XA carrying the arabinose pathway genes on multicopy vectors (Figure 1A).

The effect of the presence of the arabinose pathway on xylose utilization was also tested. TMB 3061 and TMB 3063 grew on xylose with growth rates of 0.03 h^-1 ^and 0.012 h^-1^, respectively, compared with 0.08 h^-1 ^for the parental strain TMB 3400. The decrease in xylose growth could not be explained by a decrease in the XR or XDH levels, since both arabinose-growing strains TMB 3061 and TMB 3063 had similar XR and XDH activities than the parental strain TMB 3400. The XR activities were 0.13 ± 0.01 and 0.10 ± 0.01 for TMB 3061 and TMB 3063, respectively, compared with 0.09 ± 0.00 for TMB 3400. The XDH activities were 0.52 ± 0.03 and 0.81 ± 0.08 for TMB 3061 and TMB 3063, respectively, and 0.59 ± 0.08 for TMB 3400.

### Fermentation of xylose and arabinose

**Figure 4:**
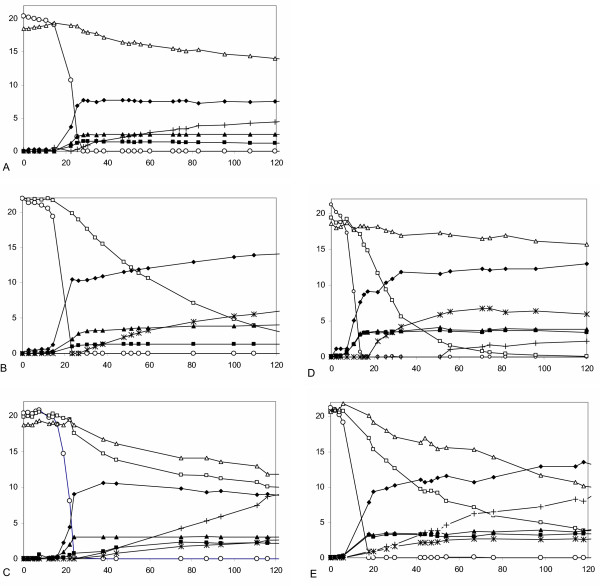
Anaerobic batch fermentations by recombinant *S. cerevisiae *in defined mineral medium with 20 g/l glucose and 20 g/l arabinose (A), 20 g/l glucose and 20 g/l arabinose (B) or 20 g/l glucose, 20 g/l xylose and 20 g/l arabinose (C-E) as carbon sources. **A**. **B**. **C**. BWY02.XA (xylose and arabinose-growing laboratory strain); **D**. TMB 3400 (xylose-growing industrial strain); **E**. TMB 3063 (xylose and arabinose-growing industrial strain). Symbols: ○ glucose; □ xylose; △ arabinose; × xylitol; + arabitol; ▲ glycerol; ◆ ethanol; ■ biomass.

Xylose and arabinose fermentation by strain BWY02.XA was independently investigated in anaerobic batch fermentation in defined mineral medium containing either 20 g/l glucose and 20 g/l xylose or 20 g/l glucose and 20 g/l arabinose (Table 2, Figure 4A–B). Glucose was included to support biomass formation, since BWY02.XA did not grow anaerobically on xylose or arabinose. Product yields were determined in the pentose phase of the fermentation, *i.e*. after glucose depletion. About 20% of the arabinose was consumed during the arabinose fermentation (Figure 4A) with a rate of 0.03 g arabinose h^-1 ^g cells^-1 ^(Table 2), whereas nearly all xylose was consumed during the xylose fermentation (Figure 4B) with a rate of 0.1 g xylose h^-1 ^g cells^-1 ^(Table 2). Ethanol was produced from xylose with the yield of 0.23 g ethanol g consumed xylose^-1^during the xylose phase of the xylose fermentation (Table 2), whereas no ethanol was produced from arabinose.

**Table 2:** Xylose and arabinose consumption rates and product yields from anaerobic batch fermentations by strains BWY02.XA (xylose and arabinose-growing laboratory strain), TMB 3400 (xylose-growing industrial strain) and TMB 3063 (xylose and arabinose-growing industrial strain). Defined mineral medium with mixtures of 20 g/l glucose and 20 g/l xylose or 20 g/l glucose and 20 g/l arabinose as well as 20 g/l glucose, 20 g/l xylose and 20 g/l arabinose were used as indicated. Product yields are calculated from the pentose phase of the fermentation. (n.a. not applicable; n.d. not detected)

**Substrate**	**Strain**	**q_xylose _** (g xylose h^-1 ^g cells^-1^)	**q_arabinose _** (g arabinose h^-1 ^g cells^-1^)	**Y_arabitol _** (g arabitol g arabinose^-1^)	**Y_xylitol _** (g xylitol g xylose^-1^)	**Y_glycerol _** (g glycerol g pentose^-1^)	**Y_acetate _** (g acetate g pentose^-1^)	**Y_ethanol _** (g ethanol g pentose^-1^)	final ethanol concentration (g l^-1^)
Glucose + xylose	BWY02.XA	0.1 ± 0.0	n.a.	n.a.	0.34 ± 0.01	0.12 ± 0.01	0.02 ± 0.00	0.23 ± 0.01	14.2 ± 0.7
Glucose + arabinose	BWY02.XA	n.a.	0.03 ± 0.00	0.77 ± 0.22	n.a.	0.00 ± 0.02	0.10 ± 0.04	n.d.	9.0 ± 2.0
Glucose + xylose + arabinose	BWY02.XA	0.041 ± 0.080	0.035 ± 0.014	1.14 ± 0.18	0.33 ± 0.03	n.d.	n.d.	0.07 ± 0.1	10.4 ± 2.2
	TMB 3400	0.066 ± 0.015	0.01 ± 0.00	approx. 1*	0.42 ± 0.00	0.01 ± 0.00	n.d.	0.09 ± 0.0	12.8 ± 0.5
	TMB 3063	0.042 ± 0.002	0.029 ± 0.002	0.68 ± 0.17	0.11 ± 0.03	0.03 ± 0.00	0.06 ± 0.01	0.16 ± 0.03	14.7 ± 2.0

(* not possible to measure due to small amounts of arabinose consumed and arabitol produced)

Co-fermentation of arabinose and xylose was studied by performing anaerobic batch fermentation using a mixture of 20 g/l glucose, 20 g/l xylose and 20 g/l arabinose as carbon sources. The laboratory strain BWY02.XA and the industrial strains TMB 3400 and TMB 3063 were tested in this set-up (Figure 4C–4E). The specific xylose and arabinose consumption rates and product yields calculated from the pentose phase of the fermentation are summarized in Table 2. Strain BWY02.XA (Figure 4C) consumed arabinose and xylose simultaneously with approximately the same consumption rates of *ca*. 0.04 g h^-1 ^g cells^-1 ^(Table 2). Nearly half of both arabinose and xylose was consumed (Figure 4C). The xylitol yield on consumed xylose was 0.33 g xylitol g xylose^-1^, whereas the arabitol yield on consumed arabinose was about 1 g arabitol g arabinose^-1 ^(Table 2).

TMB 3400 (Figure 4D) consumed nearly all available xylose (about 20 g/l) and about 3 g/l of the arabinose, whereas TMB 3063 (Figure 4E) consumed slightly less xylose (16 g/l) but much more arabinose (11 g/l). TMB 3400 and TMB 3063 had xylose consumption rates of 0.066 g xylose h^-1 ^g cells^-1 ^and 0.042 g xylose h^-1 ^g cells^-1^, respectively. The arabinose consumption rate of TMB 3400 was 0.010 g arabinose h^-1 ^g cells as compared to 0.029 g arabinose h^-1 ^g cells for TMB 3063 (Table 2). Considerable amount of xylose was consumed in the presence of glucose in both strains, whereas arabinose consumption started only after glucose depletion (Figure 4E). In both strains, ethanol was produced from pentose sugars. The final ethanol concentration was highest for the xylose- and arabinose-fermenting TMB 3063 (Table 2), despite a reduced xylose consumption rate. Reduced xylitol and arabitol formation was observed in TMB 3063 (Table 2), accompanied by improved ethanol yield from pentoses.

## Discussion

D-Xylose or L-arabinose-consuming *S. cerevisiae *strains have previously been independently developed by introduction of heterologous enzymes necessary for the assimilation of either of the sugars [6,7,10-12]. However, co-fermentation of the two pentose sugars by *S. cerevisiae *has not, to the best of our knowledge, previously been reported. We have introduced both the arabinose and the xylose pathways in *S. cerevisiae *strains to investigate the effects and possible limitations of the combined metabolism of the two pentoses. Xylose utilization was enabled by overexpression of the *P. stipitis *genes coding for XR and XDH as well as the endogenous XK through chromosomal integration. Arabinose assimilation was enabled through heterologous expression of the bacterial arabinose pathway consisting of L-arabinose isomerase (*AraA*), L-ribulokinase (*AraB*) and L-ribulose-5-P-4-epimerase (*AraD*) genes.

The combination of xylose and arabinose pathways was first tested in a laboratory strain. However, industrial fermentation of lignocellulosic substrates requires robust industrial *S. cerevisiae *strains because chemical detoxification of the industrial substrates is not economically feasible [5]. The xylose-fermenting strain TMB 3400 has been found to be inhibitor tolerant [23] and an efficient ethanol producer in undetoxified lignocellulose hydrolysate [24]. Therefore, the first industrial arabinose and xylose co-fermenting *S. cerevisiae *strain was constructed by introducing the bacterial arabinose pathway in TMB 3400 [18].

*AraB *was introduced as a single copy in both strain backgrounds to avoid the negative effects of kinase overexpression [25,26]. Since AraA and AraD were needed in high levels [11], multicopy plasmids were used for high-level expression of *AraA *and *AraD *in the laboratory strain, generating strain BWY02.XA. For the industrial strain, where plasmid usage was not applicable, a method for multiple chromosomal integration in the rDNA region was applied. The first resultant arabinose-growing strain TMB 3061 displayed limited arabinose growth compared with the strain BWY02.XA, whereas the further improved strain TMB 3063 with additional copies of the L-arabinose isomerase gene reached the growth performance of the plasmid-based laboratory strain BWY02.XA.

Co-utilization of arabinose and xylose was demonstrated with the laboratory strain BWY02.XA carrying the arabinose and the xylose pathways. BWY02.XA as well as the industrial strains TMB 3061 and TMB 3063 grew aerobically on both sugars and co-consumed both sugars simultaneously in aerobic batch culture and anaerobic batch fermentation. The simultaneous consumption of xylose and arabinose demonstrated that co-utilization of pentose sugars in *S. cerevisiae *differed from the case of glucose and xylose utilization, where glucose is usually consumed first and xylose consumption starts only when glucose is at least partially depleted [18]. Arabinose was the preferred sugar for BWY02.XA in aerobic cultivation on a mixture of xylose and arabinose, whereas xylose was the preferred sugar for TMB 3061. This is possibly due to the differences in gene copy numbers between the strains, since the laboratory strain carries the arabinose genes on multicopy plasmids, whereas the genes have been chromosomally integrated in TMB 3061.

Anaerobic fermentation of arabinose and xylose was tested both independently (Figure 4A–B) and as a mixture (Figure 4C) for the laboratory strain BWY02.XA. Nearly all xylose was consumed in the xylose fermentation, and ethanol was produced from xylose. In contrast, only 20% of the arabinose was consumed in the arabinose fermentation. The consumed arabinose was almost completely converted to arabitol. This is most likely due to the L-arabinose reductase activity of the *P. stipitis *XR [27]. XR has similar K_m _values for both xylose and arabinose [27,28]. Moreover, no arabitol formation was reported in the arabinose-fermenting strain JBY25-4M [11] that did not contain the xylose pathway enzymes. In a mixture of arabinose and xylose, the xylose consumption rate, the xylitol yield and the final ethanol concentration were reduced compared with the fermentation of xylose only. The reduced ethanol level could be seen as a consequence of the reduced xylose consumption rate observed for this strain in the presence of arabinose. The reduced xylose consumption rate may in turn have resulted from the competition of the two pentose sugars for reduction by XR. It is also possible that the capacity of the pentose phosphate pathway is limiting pentose consumption. In addition, there may be competition for the unspecific pentose transporters in *S. cerevisiae *[29]. For example, Gal2p has been shown to transport L-arabinose [30] as well as D-xylose [29]. Thus, further improvement should be possible by overexpressing the unspecific transporter *GAL2 *[11], which is known to improve arabinose utilization, or by overexpressing a specific transporter for either xylose [31] or arabinose.

Introduction of the arabinose pathway in the industrial strain TMB 3400 resulted in reduced growth on xylose in strains TMB 3061 and TMB 3063, which could not be explained by decreased activities of the xylose pathway enzymes XR and/or XDH. Instead, we believe that the reduced growth results from the metabolic load observed when expressing multiple heterologous enzymes at high levels [32].

The industrial strain TMB 3063 had an improved fermentation performance compared to the parental strain TMB 3400 in fermentation of mixture of glucose, xylose and arabinose. Arabinose and xylose were consumed simultaneously under anaerobic conditions by TMB 3063 and about half of the available arabinose was now used. The ethanol yield from pentose was nearly doubled in TMB 3063, and the final ethanol concentration was increased by 15% compared to TMB 3400, despite the reduced xylose consumption of TMB 3063. The improvement in ethanol yield was accompanied by a decrease in xylitol and arabitol yields in TMB 3063. The decrease in xylitol formation in TMB 3063, as well as in the laboratory strain, is possibly a result of the competing reduction of L-arabinose by XR, which could result in larger fraction of xylose being reduced with NADH. Thus, additional genetic modification of the xylose and arabinose utilization pathways to optimize the amounts of the heterologous enzymes expressed could improve the co-fermentation of xylose and arabinose.

## Conclusion

The novel arabinose- and xylose-growing strains displayed arabinose and xylose co-consumption under both aerobic and anaerobic conditions. The functional co-consumption of arabinose and xylose was demonstrated with laboratory strain BWY02.XA. Industrial xylose- and arabinose-growing strains TMB 3061 and TMB 3063 were constructed, of which TMB 3063 reached arabinose growth performance similar to BWY02.XA. Under anaerobic conditions, TMB 3063 consumed highest amount of arabinose, however, with arabitol was a considerable by-product. At the same time, the consumption of arabinose reduced the xylitol yield by 70%, which resulted in an overall increase in ethanol formation. The results show that arabinose and xylose can be co-consumed by recombinant *S. cerevisiae*, but efficient ethanolic fermentation of L-arabinose will require further genetic modification.

## Materials and methods

### Strains and media

Plasmids and yeast strains used in this study are summarized in Table 1. *E. coli *strain DH5α (Life Technologies, Rockville, MD, USA) was used for cloning and grown in LB-medium [33] with 100 mg/l ampicillin or 40 mg/l kanamycin. In aerobic batch cultivations, *S. cerevisiae *was grown in Yeast Nitrogen Base medium (YNB) (6.7 g/l Difco Yeast Nitrogen Base without amino acids; Becton, Dickinson and Company, Sparks, MD, USA) supplemented with 20 g/l glucose, 50 g/l xylose or 50 g/l arabinose as carbon sources and buffered at pH 5.5 with 10.21 g/l potassium hydrogen phthalate. When the sugar concentration in the medium exceeded 20 g/l, the concentration of YNB was doubled. In anaerobic fermentations, defined mineral medium [34] was used, supplied with 0.4 g/l Tween 80 and 0.01 g/l ergosterol and 20 g/l glucose, 20 g/l xylose and/or 20 g/l arabinose as carbon sources. Plate cultures were made on YPD (10 g/l yeast extract, 20 g/l peptone, 20 g/l glucose, 20 g/l agar) or YNB (6.7 g/l Difco YNB, 30 g/l agar, 20 g/l glucose, 50 g/l arabinose or 50 g/l xylose). When needed, 200 mg/l geneticin (Life Technologies, Rockville, Md.) was added to YPD plates.

### Molecular biology techniques

Standard molecular biology techniques were used [33]. The calcium chloride method was used for bacterial transformation [35] and the lithium acetate method was used for yeast transformation [36]. Plasmids were isolated from bacteria with Quantum Prep kit (Bio-Rad, Hercules, USA). Yeast chromosomal DNA was extracted with Easy-DNA Kit (Invitrogen, Groningen, The Netherlands). DNA sequencing was performed with Abi-Prism BigDye cycle sequencing kit (Applied Biosystems, Weiterstadt, Germany). PCR was performed with the following program: 95°C for 5 min, 45 cycles of 95°C for 30 s, 50°C for 30 s and 72°C for 1 min 20 s and 72°C for 7 min. Taq-polymerase (Fermentas, Vilnius, Lithuania) was used in analytical PCR and PWO polymerase (Roche Diagnostics AB, Bromma, Sweden) in preparative PCR. T4-nucleotide ligase and restriction enzymes were purchased from Fermentas (Vilnius, Lithuania).

### Plasmid construction

Plasmid YEpURAaraA (Table 1) was constructed by subcloning the *B. subtilis araA *gene flanked by the truncated *HXT7 *promoter [37] and the *CYC1 *terminator from plasmid YEp*araA *[11] into plasmid p426H7 [11].

For the construction of an integrative vector carrying the mutated *E. coli AraB *gene [11], the KanMX antibiotic marker [38] was first cloned into the plasmid YEp*araB *[11] which already contained the *AraB *gene. A fragment with the KanMX-marker flanked by *loxP*-sequences was amplified from plasmid pUG6 [38] with primers containing the *ApaI *restriction site and cloned in YEp*araB *[11]. The 2 μ-sequence and the ampicillin resistance marker in the resultant plasmid were removed by cutting with *Eco105I *and *Eco31I *followed by self-ligation of the plasmid, resulting in plasmid YIpAraB (Table 1, Figure 2A). The *E. coli *colonies carrying the plasmid were selected for kanamycin resistance.

### Construction of the template plasmids prDNAAraA and prDNAAraD

To enable cloning of the bacterial *AraA *and *AraD *genes in between flanking sequences of *S. cerevisiae *rDNA sequence, a plasmid containing two adjacent regions from the *S. cerevisiae *rDNA was constructed. Two PCR-products were amplified from *S. cerevisiae *CEN.PK 113-5D rDNA *NTS2 *region [39] with primers containing restriction sites for *KpnI *(5'-AGTCGGTACCGATATGGAATGGTTGGCGAAG-3') and *ApaI *(5'-TCAGCCCGGGTGGCTTCCTATGCTAAATCCC-3') or *SacI *(5'-AGTCGAGCTCTGTTAAGTATACATGTATATATTGC-3') and *SacII *(5'-AGTCCCGCGGCGTTGCAAAGATGGGTTGAAAG-3'). The ~500 bp long PCR products were digested with the corresponding restriction enzymes and cloned sequentially into pBluescript SK-(Stratagene, La Jolla, CA, USA). The resulting plasmid was named prDNA2 (Figure 2B).

*AraA *and *AraD *genes were independently cloned between the rDNA sequences of prDNA2 to create a template for obtaining PCR-products consisting of either of the genes flanked by the rDNA sequences. Fragments containing *AraA *or *AraD *gene, flanked by the truncated *HXT7*-promoter [37] and the *CYC1*-terminator, were digested from plasmids YEp*araA *or YEp*araD *[11] with *Eco52I *and *ApaI*. The fragments were cloned into prDNA2, resulting in plasmids prDNAAraA and prDNAAraD (Figure 2B), respectively.

### Aerobic cultivation

Pre-cultures were grown until late exponential phase in YNB medium with 20 g/l glucose except for strain BWY02.XA, which was grown on 20 g/l arabinose. Cells were washed with sterile water and inoculated at an OD_620 nm _of 0.2 in YNB medium with either 50 g/l arabinose, 50 g/l xylose, 20 g/l glucose, or a mixture of 25 g/l arabinose and 25 g/l xylose. 50 ml cultures were grown in 500 ml baffled shake flasks at 30°C with 180 rpm agitation in a shaker incubator (Gallenkamp INR-200, Leicester, UK). The growth rates were measured at least in duplicate with standard deviations of less than 10%.

### Anaerobic batch fermentation

Anaerobic batch fermentation was performed in Biostat^® ^bioreactors (B. Braun Biotech International, Melsungen, Germany). Precultures were performed as described above. Different combinations of 20 g/l glucose, 20 g/l xylose and/or 20 g/l arabinose were used as carbon sources. The fermentations were inoculated at an OD_620 nm _of about 0.2, run at 30°C, with 200 rpm stirring and pH 5.5 maintained by addition of 3 M KOH. Anaerobic conditions were maintained by sparging with nitrogen gas (containing less than 5 ppm O_2_, AGA, Malmö, Sweden) with the flow rate of 0.2 l min^-1 ^controlled with a mass flow-meter (Bronkhorst, HI-TECH, Ruurlo, The Netherlands). Carbon dioxide and oxygen concentrations in the exhaust gas were measured with a Carbon Dioxide and Oxygen Monitor type 1308 (Bruel & Kjaer, Copenhagen, Denmark) in order to follow the course of the fermentation and verify anaerobiosis. The experiments were performed in duplicate.

### Analyses

Concentrations of glucose, xylose, glycerol, and acetic acid were analyzed by HPLC (Beckman Instruments, Fullerton, CA, USA) using three in series connected Aminex HPX-87H ion exchange columns (Bio-Rad, Hercules, USA). The mobile phase was 5 mM H_2_SO_4_, temperature 45°C and the flow rate 0.6 ml/min. The concentrations of arabinose, arabitol and xylitol were analyzed with HPLC (Waters, Milford, Massachusetts, USA) using a HPX-87P (Bio-Rad, Hercules, USA) ion exchange column at 85°C with water as the mobile phase at a flow rate of 0.5 ml/min. Refractive index detector (RID-6A, Shimadzu, Kyoto, Japan) was used for quantification in both HPLC systems. The concentration of ethanol was calculated from the degree of reduction balance due to evaporation of the produced ethanol from the reactors. Triplicate measurements of cell dry weight were performed by filtering a known volume of the culture through a pre-weighed nitrocellulose filter with 0.45 μm pores. The filters were dried in a microwave oven and weighed.

### XR and XDH activity

Cell free extracts were made from cells grown on YNB medium containing glucose. The cells were harvested in exponential growth phase, washed with sterile water and treated with yeast protein extraction reagent Y-PER according to the manufacturer's protocol (Pierce, Rockford, IL, USA). Protein concentrations were determined with Micro-BCA™ kit (Pierce, Rockford, IL, USA) using COBAS MIRA plus (Roche, Mannheim, Germany) automatic analyzer. XR and XDH activities were determined as previously described [40,41].

## Contributions of authors

KK constructed and characterized the industrial strains, participated in the design of the industrial strains, performed data interpretation and drafted the manuscript.

BW constructed and characterized the laboratory strains.

EB participated in design of the study, design of the laboratory strains and commented the manuscript.

BHH participated in design of the study and commented the manuscript.

MFGG participated in design of the study, participated in the design of the industrial strains, performed data interpretation and assisted in drafting the manuscript.

All authors have read and approved the manuscript.
